# Contextual property detection in Dutch diagnosis descriptions for uncertainty, laterality and temporality

**DOI:** 10.1186/s12911-021-01477-y

**Published:** 2021-04-07

**Authors:** Eva S. Klappe, Florentien J. P. van Putten, Nicolette F. de Keizer, Ronald Cornet

**Affiliations:** Department of Medical Informatics, Amsterdam Public Health Research Institute, Amsterdam UMC, University of Amsterdam, Meibergdreef 15, 1105AZ Amsterdam, The Netherlands

**Keywords:** Electronic health record, Problem list, Problem-oriented medical record, Rule-based algorithm development, Single-center and multicenter validation, Reuse of clinical data

## Abstract

**Background:**

Accurate, coded problem lists are valuable for data reuse, including clinical decision support and research. However, healthcare providers frequently modify coded diagnoses by including or removing common contextual properties in free-text diagnosis descriptions: uncertainty (*suspected glaucoma*), laterality (*left glaucoma*) and temporality (*glaucoma 2002*). These contextual properties could cause a difference in meaning between underlying diagnosis codes and modified descriptions, inhibiting data reuse. We therefore aimed to develop and evaluate an algorithm to identify these contextual properties.

**Methods:**

A rule-based algorithm called UnLaTem (Uncertainty, Laterality, Temporality) was developed using a single-center dataset, including 288,935 diagnosis descriptions, of which 73,280 (25.4%) were modified by healthcare providers. Internal validation of the algorithm was conducted with an independent sample of 980 unique records. A second validation of the algorithm was conducted with 996 records from a Dutch multicenter dataset including 175,210 modified descriptions of five hospitals. Two researchers independently annotated the two validation samples. Performance of the algorithm was determined using means of the recall and precision of the validation samples. The algorithm was applied to the multicenter dataset to determine the actual prevalence of the contextual properties within the modified descriptions per specialty.

**Results:**

For the single-center dataset recall (and precision) for removal of uncertainty, uncertainty, laterality and temporality respectively were 100 (60.0), 99.1 (89.9), 100 (97.3) and 97.6 (97.6). For the multicenter dataset for removal of uncertainty, uncertainty, laterality and temporality it was 57.1 (88.9), 86.3 (88.9), 99.7 (93.5) and 96.8 (90.1). Within the modified descriptions of the multicenter dataset, 1.3% contained removal of uncertainty, 9.9% uncertainty, 31.4% laterality and 9.8% temporality.

**Conclusions:**

We successfully developed a rule-based algorithm named UnLaTem to identify contextual properties in Dutch modified diagnosis descriptions. UnLaTem could be extended with more trigger terms, new rules and the recognition of term order to increase the performance even further. The algorithm’s rules are available as additional file 2. Implementing UnLaTem in Dutch hospital systems can improve precision of information retrieval and extraction from diagnosis descriptions, which can be used for data reuse purposes such as decision support and research.

**Supplementary Information:**

The online version contains supplementary material available at 10.1186/s12911-021-01477-y.

## Background

The problem-oriented medical record—a structured organization of patient information per provided medical problem—is successful in helping healthcare providers to get a good understanding of the temporality of patients [1–3]. A core element is the problem list, which presents a list of active and inactive diagnoses relevant to current care of the patient [2]. Problem lists support reuse of data to e.g. trigger rules of a decision support system, create patient cohorts for quality registries or medical research [4, 5]. However, in order to realize these benefits, problem lists need to be accurate and complete, and problem list entries should be coded [4–7].

Although healthcare providers acknowledge the importance of accurate problem lists [8–10], problem lists often remain inaccurate and incomplete [11–14]. Healthcare providers consider free-text documentation typically as important, because they have concerns that by recording structured data on the problem list important information could be omitted [15]. As a consequence, when healthcare providers do code diagnoses on the problem list, they may modify the description of these diagnoses or add details in free-text fields as they often find the default diagnosis description insufficient or because they cannot find the diagnosis they are looking for [4, 16].

The context of the diagnoses in modified descriptions is crucial for determining the clinical status of a patient [17–20]. Previous research showed that healthcare providers describe levels of certainty in clinical free text, e.g., to specify a working diagnosis [16, 20–24]. Certainty levels vary from affirmed (certain) to non-affirmed (uncertain) levels of speculation. Uncertainty can be defined as the expressions of hypotheses, tentative conclusions, and speculations [24]. Uncertainty described in diagnosis descriptions could indicate a change in the meaning of a modified description compared to the default description. For instance, if the default diagnosis description for a code is *glaucoma* but the modified description *suspected glaucoma*, the problem list indicates by its code that the patient has glaucoma, while the description indicates that this diagnosis is not yet confirmed. Consequently, if researchers select all patients who suffered from glaucoma, the system returns all patients with the diagnosis code for *glaucoma*, although some patients might have had *suspected* glaucoma*.*

Next to uncertainty it is also important to know if a diagnosis is specified with laterality (e.g. *left* or *right* glaucoma) because missing laterality could lead to medical errors, such as procedures being performed on the wrong extremity [25]. Problems that should be specified by laterality are not always available in clinical terminologies, which therefore requires adding laterality in the description [26]. Furthermore, healthcare providers may argue that listing temporality is important (e.g. glaucoma *2002)*, explaining the timeline of a disease, symptom or event [23, 27]. Specifying a diagnosis with temporality could also be useful for prompting more frequent testing, for instance for breast cancer [28].

Again, it is important to identify temporality, because temporality described in a modified description indicates that the problem is a former problem, while the code indicates it is a current problem [17, 27, 29–32]. The examples given above might result in discrepancies between codes and modified descriptions or other free text which might lead to inappropriate care or research findings [5, 33–38]. The identification of context of information in terms of uncertainty, laterality and temporality is therefore an important task [17, 21, 35, 39]. Uncertainty, laterality and temporality can be referred to as contextual properties, because the information is not captured in the diagnosis itself, but provides the context of the diagnosis [17].

Several algorithms have been developed and evaluated to identify contextual properties in clinical free text [5, 7, 17, 27, 32]. These algorithms can extract concepts from free text and map these concepts to a standardized vocabulary [6], such as the tools MetaMap [40] and IndexFinder [41]. Additionally, regular expressions can be used to identify specific contextual properties. For instance NegEx is an algorithm that uses regular expressions to identify negated (i.e. ‘ruled-out’) diagnoses [32]. ConText, an algorithm that was developed based on NegEx [32], identifies several contextual properties in clinical free text, including whether a condition is negated, but also hypothetical, historical, or experienced by someone else [17]. However, techniques like ConText have been developed for English text, and few algorithms can identify contextual properties in other languages, such as Dutch [27, 42]. One example is ContextD, which identifies the same contextual properties as ConText, but for Dutch [27]. For the historical values of the temporality property, performance ranged from 26 to 54%. To our knowledge, no algorithms have been developed to recognize laterality or uncertainty in Dutch text.

The purpose of this study was to develop and evaluate a new algorithm to be called UnLaTem (uncertainty, laterality, temporality) for identifying (removal of) uncertainty, laterality and temporality in modified diagnosis descriptions. These properties should be identified before reusing diagnosis data as they could cause a difference in meaning between codes and descriptions. We applied the algorithm to Dutch free-text modified diagnosis descriptions, to gain insights into the extent to which diagnosis descriptions contain (removal of) uncertainty, laterality and temporality.

## Methods

### Dataset

In most Dutch hospitals, the interface terminology underlying the problem list in the EHR systems is the Diagnosis Thesaurus (DT), provided by Dutch Hospital Data (DHD), which is mapped to ICD-10 and SNOMED CT [43]. The DT is used by healthcare providers to select the best-fitting code for their patients’ problems.

An anonymized dataset was extracted from the EHR system (Epic) of the Amsterdam University Medical Center (UMC). This dataset included all diagnoses recorded on the problem list with the DT and their descriptions, of all non-shielded patients (e.g. VIP-patients are shielded) admitted to the hospital in 2017. To develop and validate the algorithm, we selected all records in which the free-text field ‘description’ differed from the default diagnosis description. This included complete replacements (e.g. *glaucoma* changed to *hypertension*), additions (*e.g. glaucoma* changed to *suspected glaucoma*) and removals (e.g. *suspected glaucoma* changed to *glaucoma*). Thus, only exact matches were not included. A multicenter dataset including data from five anonymous Dutch hospitals, all using the same EHR (Epic), was used for second validation of the algorithm. According to the supplier of the multicenter dataset, a total of 1,035,059 diagnoses were registered for these five hospitals between April 2018 and May 2019. Note that the multicenter dataset also contained records from Amsterdam UMC, but these covered a different time frame than the records of the single-center dataset. In contrast to the Amsterdam UMC dataset, this multicenter dataset was constructed to include only encoded problems for which the problem description was modified by the end-user (n = 175,210). Further characteristics of the two datasets are shown in Table 1.

**Table 1:** Characteristics of the single-center and multicenter dataset

Dataset/characteristics	Single-center dataset: Amsterdam UMC	Multicenter dataset: five Dutch hospitals
Total records available, n	288,935	1,035,059
Modified descriptions, n(%)	73,280 (25.4)	175,210 (16.9)
Time period	1-1-2017–31-12-2017	28-4-2018–29-5-2019
Medical specialties, n	37	62 original; 41 after clustering
Usage for this study	Development and internal validation of algorithm	Multicenter validation of algorithm and to measure the frequency of types of contextual properties

Both datasets included four variables: an ICD-10 code, default diagnosis descriptions, modified descriptions and the medical specialty that modified the diagnosis description. For example, ICD-10 code *I10* has diagnosis description *essential hypertension.* A modified free-text description could be *suspected hypertension.* For the multicenter dataset, we combined the specialties that were related, such as Audiology and Audiological center (see Appendix 1) [44] after which 41 specialties remained**.**

### Data selection

Of the 73,280 modified descriptions in the Amsterdam UMC dataset, 54,960 (75%) records were used for development of the algorithm. Of the remaining 18,320 (25%), we randomly selected 1000 records for validation while removing case-insensitive duplicate combinations of diagnosis descriptions and modified descriptions. The remaining 17,320 records could be used for future development and validation of the algorithm. Similarly, a second validation of the algorithm was conducted with 1000 records of the 175,210 modified descriptions of the multicenter dataset.

### Development of the algorithm UnLaTem

**Fig. 1:**
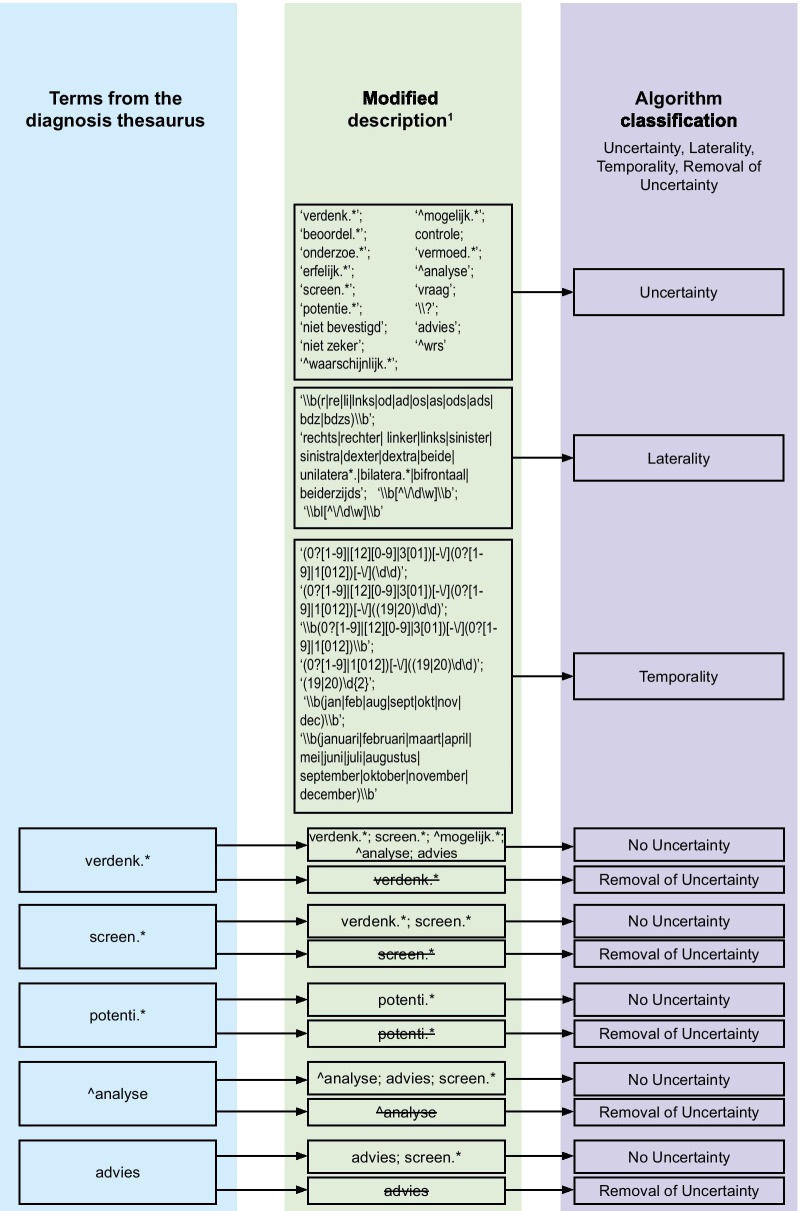
Algorithm regular expressions and corresponding categories. The rectangles in the first and second column contain the regular expressions. the rectangles in the third column contain the properties that result from the inclusion of the regular expressions. ^1^Modified descriptions can be classified according to multiple contextual properties (uncertainty, laterality and/or temporality)

We developed an algorithm that uses regular expressions to identify whether a modified diagnosis description contained (removal of) uncertainty, laterality and/or temporality. In this study, we treat uncertainty as expressions of belief, including at least one tentative conclusion or speculative fragment described in diagnosis descriptions [24]. The detection of uncertainty addition and removal was based on regular expressions that therefore describe tentative conclusions, speculation and hypotheses in diagnoses. Temporality detection was based on regular expressions indicating various forms of dates and laterality was based on regular expressions that indicate left and right in various forms. To discover all variations of the contextual properties, two authors (ESK and FJP) manually checked all descriptions in the development set (n = 54,960). To identify ‘removal’, we also manually checked whether uncertainty and laterality occurred in the unique default diagnosis descriptions of the DT (n = 10,936). Based on all found variations, regular expressions were established. For instance, laterality could be described as *left* and *right,* but also *le* and *ri* (Dutch: *links* and *rechts*, *li* and *re*). A final set of regular expressions was made available for all three properties, which is shown in Fig. 1. The translation of the terms to English can be found as Additional file 1. The algorithm was developed in R using R.Studio v 1.2.1335 for Windows.

### Validation and performance of the algorithm

The single-center and multicenter validation sets were independently manually annotated by the same two authors (ESK and FJP). The annotators distinguished the four contextual properties based on guidelines provided by ESK explaining the process and each of the properties in detail with examples. We reported the interrater reliability score and Cohen’s kappa score to examine agreement between annotators [45]. After independent annotation, both annotators compared their results and discussed the annotations until they reached consensus on discrepancies. After agreement, the sets were used as reference standard.

Confusion matrices were used to determine the performance, i.e. the agreement between the manual expert-based outcome and the algorithm’s outcome per type of property in both validation sets. An example of a confusion matrix is shown in Appendix 2A. Based on these matrices, we determined the actual prevalence, which is defined as the definitive percentage of identified properties (i.e. false negatives and true positives). We calculated the performance of UnLaTem in terms of recall, specificity and precision for both validation samples [46, 47]. Recall is defined as the proportion of modified descriptions that was correctly identified with the correct type(s) of contextual properties (i.e., true positives). The specificity is defined as the proportion of modified descriptions that were correctly identified not to have type(s) of properties (i.e., true negatives). The precision is the probability that the algorithm correctly identified the type(s) of properties in the modified descriptions.

We determined the mean values of the recall, specificity, prevalence and precision of the two validation samples by using the inverse variance-weighted average method [48, 49]. This method calculates the weighted mean of the two validations samples, by taking into account the standard errors of the recall, specificity and prevalence. Haldane-Anscombe correction was applied to calculate an approximation of the weighted means, i.e., if a confusion matrix contained zeros, we added 0.5 to all counts [50–52]. Appendix 2A and 2B show all formulas [48, 52, 53].

### Error analysis

In order to assess the adequacy of the rule-based approach for contextual property assignment in Dutch modified diagnosis descriptions, we analyzed all incorrect assignments and placed them in an error class, i.e. the counts of the total number of false positives and false negatives. In line with ConText, four error classes were distinguished [17]. The classes included Missing terms (error can be prevented by adding trigger terms, pseudo-trigger terms or termination terms to UnLaTem’s term lists), Simple extension (error can be prevented by adding another rule to the algorithm), Outside framework (prevention of error requires a change that cannot be accommodated within current UnLaTem’s framework) and Annotation/implementation (error is the result of a mistake in the reference standard annotations or a bug in the implementation of the algorithm).

### Application of the algorithm

UnLaTem was used to determine the apparent prevalence of (removal of) uncertainty, laterality and/or temporality within the modified descriptions of the Amsterdam UMC dataset (n = 73,280) and the multicenter dataset (n = 175,210). Based on the apparent prevalence, we calculated the actual prevalence using the Rogan–Gladen estimator, of which the formula is shown in Appendix 2C [54]. Note that this is the actual prevalence of contextual properties within the modified descriptions, and not within the complete datasets. The actual prevalence was reported per specialty in the multicenter dataset.

## Results

### Development of the algorithm

The regular expressions and trigger terms for (removal of) uncertainty, laterality and temporality for the algorithm are shown in Fig. 1, in the second column. For example, if the default diagnosis description was *glaucoma* and the modified description *glaucoma screening*, the algorithm’s uncertainty-value was True. If the default diagnosis description was *suspected glaucoma* and the modified description included *suspected*, *screening*, *possible*, *analysis* or *advice*, the algorithm returned False for uncertainty. That is, because both expressions indicate that having glaucoma was suspected. If the default diagnosis description was *suspected glaucoma* and the modified description *glaucoma*, the algorithm returned True for removal of uncertainty. Note that not all default diagnoses available in the DT also contained a version with pre-coded uncertainty. For instance, *glaucoma* and *suspected glaucoma* exist as default diagnosis descriptions but *hypertension* and the corresponding *suspected hypertension* do not.

Only a few records that included removal of laterality were retrieved. However, these modified descriptions were specifications or generalizations of the diagnosis term. For instance, *left sided heart failures* was changed to *decompensatio cordis*, the Latin name for heart failure. We considered these modifications another type of property, which was not included in this version of the algorithm.

Also, note that one modified description can have multiple properties (*2015 left* eye infection is categorized as both laterality as well as temporality for the default description *eye infection*).

### Validation and performance of the algorithm

In both validation sets (n = 1000 + 1000), we discovered that terms from modified descriptions were identical to default diagnosis descriptions, but were flagged as ‘modified’ because some descriptions included spaces or because the healthcare provider had clicked in the description field. We removed these records after which 980 remained in the internal validation set and 996 remained in the multicenter dataset. The interrater reliability and kappa score between the two raters that defined the reference standard validation sets were determined per property and are shown in Table 2. Please note that all disagreements were solved after the initial scoring.

**Table 2:** Interrater reliability and kappa scores for the internal (n = 980) and multicenter (n = 996) validation set

Property (n)/dataset	Interrater reliability (%)^a^		Kappa score	
	Internal validation set (n = 980)	Multicenter validation set (n = 996)	Internal validation set (n = 980)	Multicenter validation set (n = 996)
Laterality	98.0 (N_ESK_ = 245, N_FJP_ = 233)	98.1 (N_ESK_ = 288, N_FJP_ = 293)	0.94	0.95
Temporality	97.7 (N_ESK_ = 163, N_FJP_ = 157)	98.1 (N_ESK_ = 96, N_FJP_ = 85)	0.91	0.88
Uncertainty	96.1 (N_ESK_ = 135, N_FJP_ = 107)	97.5 (N_ESK_ = 98, N_FJP_ = 87)	0.82	0.85
Removal of uncertainty	98.3 (N_ESK_ = 11, N_FJP_ = 14)	99.3 (N_ESK_ = 8, N_FJP_ = 11)	0.36	0.63

Descending on Kappa scores

^a^N_ESK_ is the sum of the records in the corresponding property according to annotator ESK and N_FJP_ is the sum of the records in the corresponding property according to annotator FJP

Table 3 shows the actual prevalence, which was determined using the reference standards and the recall, specificity and precision of UnLaTem to identify the properties for the validation samples. The confusion matrices are shown in Appendix 3. In addition, Table 3 shows the inverse variance-weighted averages of the recall, specificity and prevalence. We applied the weighted prevalence, recall and specificity to determine the weighted precision. Appendix 2B shows the formulas we used and the numbers for these calculations.

**Table 3:** Actual prevalence, recall, specificity and precision of all four properties in the internal (n = 980) and multicenter validation set (n = 996)

Property	Actual prevalence (%) (95% CI)			Recall (95% CI)			Specificity (95% CI)			Precision (95% CI)		
	Internal validation set	Multicenter validation set	Weighted mean (%)	Internal validation set	Multicenter validation set	Weighted mean (%)	Internal validation set	Multicenter validation set	Weighted mean (%)	Internal validation set	Multicenter validation set	Weighted mean (%)
Laterality^a^	25.3 (22.6–28.2)	29.2 (26.4–32.1)	27.4 (24.7–30.1)	100 (98.5–100)	99.7 (98.1–100)	99.9 (99.4–100)	99.0 (98.1–99.6)	97.2 (95.7–98.2)	98.6 (97.9–99.2)	97.3 (94.4–98.9)	93.5 (90.2–96.0)	96.1 (95.3–97.0)
Temporality	16.9 (14.6–19.4)	9.4 (7.7–11.4)	12.4 (7.1–17.7)	97.6 (93.9–99.3)	96.8 (91.0–99.3)	97.4 (95.3–99.4)	99.5 (98.7–99.9)	98.9 (98.0–99.5)	99.3 (98.9–99.7)	97.6 (93.9–99.3)	90.1 (82.5–95.1)	96.0 (95.2–96.9)
Uncertainty	11.9 (10.0–14.1)	10.2 (8.4–12.3)	11.0 (9.8–12.2)	99.1 (95.3–100)	86.3 (78.0–92.3)	98.1 (96.1–100)	98.5 (97.4–99.2)	98.8 (97.8–99.4)	98.6 (98.1–99.2)	89.9 (83.4–94.5)	88.9 (81.0–94.3)	89.4 (88.1–90.8)
Removal of uncertainty^a^	0.9 (0.4–1.7)	1.4 (0.8–2.3)	1.2 (0.8–1.6)	100 (66.4–100)	57.1 (28.9–82.3)	90.4 (78.5–100)	99.4 (98.7–99.8)	99.9 (99.4–100)	99.8 (99.6–100)	60.0 (32.3–83.7)	88.9 (51.8–99.7)	80.6 (78.9–82.2)

The numbers are calculated using the reference standard

^a^Removal of Uncertainty and Laterality both contained zeros in the confusion matrix

### Error analysis

Tables 4 and 5 show the results of the error analysis in the internal validation set (n = 980) and multicenter validation set (n = 996).

**Table 4:** Error analysis of false positives (FP) and false negatives (FN) in the internal validation set (n = 980)

	Total = FP + FN	Missing terms (%)	Simple extension (%)	Outside framework^a^ (%)	Annotation / implementation (%)
Laterality	7 = 7 + 0	0 (0.0)	6 (85.7)	0 (0.0)	1 (14.3)
Temporality	8 = 4 + 4	4 (50.0)	3 (37.5)	1 (12.5)	0 (0.0)
Uncertainty	14 = 13 + 1	2 (14.3)	8 (57.1)	4 (28.6)	0 (0.0)
Removal of Uncertainty	6 = 6 + 0	0 (0)	5(8.3)	0 (0.0)	1 (16.7)
Total	35 = 30 + 5	7 (20.0)	22 (62.8)	5 (14.3)	1 (2.9)

^a^Outside framework consisted only of errors that related to term order

**Table 5:** Error analysis of false positives (FP) and false negatives (FN) in the multicenter validation set (n = 996)

	Total = FP + FN	Missing terms (%)	Simple extension (%)	Outside framework^a^ (%)	Annotation / implementation (%)
Laterality	21 = 20 + 1	1 (4.8)	19 (90.4)	0 (0.0)	1 (4.8)
Temporality	13 = 10 + 3	3 (23.1)	7 (53.8)	1 (7.7)	2 (15.4)
Uncertainty	25 = 11 + 14	6 (24.0)	7 (28.0)	8 (32.0)	4 (16.0)
Removal of Uncertainty	7 = 1 + 6	3 (42.9)	1 (14.3)	0 (0.0)	3 (42.8)
Total	66 = 42 + 24	16 (24.3)	34 (51.5)	9 (13.6)	7 (10.6)

^a^Outside framework consisted only of errors that related to term order

### Application of UnLaTem

Table 6 shows the apparent and actual prevalence of the contextual properties which was determined within the modified descriptions for both the Amsterdam UMC dataset (n = 73,280) and multicenter dataset (n = 175,210).

**Table 6:** Number and percentages of the properties identified by the algorithm in the modified descriptions per dataset

Property/dataset	Amsterdam UMC dataset (n = 73,280)		Multicenter dataset (n = 175,210)		Amsterdam UMC dataset (n = 73,280)		Multicenter dataset (n = 175,210)	
	N	Apparent prevalence % (95% CI)	N	Apparent prevalence % (95% CI)	N	Actual prevalence % (95% CI)	N	Actual prevalence % (95% CI)
Laterality	17,656	24.1 (23.8–24.4)	54,081	30.9 (30.7–31.1)	17,934	24.5 (24.2–24.8)	54,931	31.4 (31.1–31.6)
Uncertainty	11,834	16.1 (15.9–16.4)	16,779	9.6 (9.4–9.7)	12,235	16.7 (16.4–17.0)	17,347	9.9 (9.5–10.3)
Temporality	10,758	14.7 (14.4–14.9)	16,582	9.5 (9.3–9.6)	11,130	15.2 (14.9–15.4)	17,156	9.8 (9.7–9.9)
Removal of uncertainty	677	0.9 (0.9–1.0)	1,991	1.1 (1.1–1.2)	751	1.0 (1.0–1.1)	2,208	1.3 (1.2–1.3)

Ordered by descending percentages of contextual properties

Appendix 4 shows actual prevalence of the contextual properties within the modified descriptions per specialty in the multicenter dataset. From the specialties with more than one thousand diagnosis descriptions, clinical genetics had the highest percentage of uncertainty within the modified descriptions (5287/5655, 93.5%), audiology had the lowest percentage of uncertainty within the modified descriptions (4/1127, 0.4%). Laterality additions were found highest in ophthalmology (13,471/16,901, 79.7%) and lowest in audiology (133/1127, 11.8%). Temporality additions were highest for internal medicine (4822/30,457, 15.8%) and lowest for audiology (0/1127, 0.0%). Removal of uncertainty was highest for urology (49/1306, 3.8%) and lowest for audiology (0/1127, 0.0%).

The most-frequent modification was adding *od* (right eye) to *cataract*, which occurred 1,237 (0.7%) times in the dataset (n = 175,210), followed by adding *os* (left eye) to *cataract,* which occurred 1,191 (0.7%) times.

## Discussion

In this study, we developed and evaluated an algorithm called UnLaTem to identify (removal of) uncertainty, laterality and temporality in Dutch modified diagnosis descriptions. The evaluation showed high performance measures, indicating good performance of the algorithm*.* Removal of uncertainty had lower performance scores, indicating that for removal of uncertainty the algorithm could be further improved. Additionally, we determined the prevalence of the contextual properties in the modified descriptions, in total and per specialty. As expected, the percentages of laterality modifications that were considerably higher than the mean value occurred in specialties for which adding laterality is important, namely ophthalmology and orthopedics. For temporality, a specialty with a considerably higher value than the mean was emergency care. This can be explained by the fact that the treating healthcare provider is usually reliant on the medical history of the patient. The percentage uncertainty was highest in clinical genetics. This can be expected as it is a specialty that provides screening service and therefore deals with high uncertainty in (initial) diagnoses. The variations of contextual properties between these and other medical specialties should be looked into in future research.

One could argue why healthcare providers have to record diagnoses on a problem list in a coded way, with the risk of adding contextual properties in free-text. In principle, natural language processing (NLP) algorithms can be used to identify diagnoses in clinical free text [55]. NLP could automatically transform clinical text into structured data that can guide clinical decision-making and data reuse such as research. However, current NLP algorithms cannot accurately identify diagnoses in Dutch or English clinical free text yet [56]. Hence, UnLaTem could be beneficial for the identification of discrepancies in modified diagnosis descriptions in Dutch.

### Strengths and limitations

A strength of this study is that we developed a successful algorithm for recognizing four contextual properties in Dutch descriptions, as it showed high performance scores in both validation sets. Furthermore, only few algorithms have been developed for the Dutch language [27, 57]. Following application of UnLaTem to the multicenter dataset, we were also able to gain insights into the correctness of underlying diagnosis codes. Identification of differences between codes and modified descriptions is essential, because reuse of problem list data relies often on coded data alone [5, 58]. The results showed for instance that a relatively high number of modified descriptions (n = 17,347, 9.9%, CI: 9.5–10.3) contained uncertainty in the multicenter dataset. Subsequently, identification of patients with a certain disease, design of disease registers or assessment of quality of care based on coded diagnoses may be error-prone [59, 60].

Our research also has several limitations. Although the overall performance of UnLaTem was high, performance was only determined for identification of contextual properties of diagnoses that were registered on the problem list, but problem list incompleteness is a widespread issue [4, 6]. We expect to find more information on diagnoses in other free-text fields, such as history notes, discharge letters and medication letters. This is important because although ‘modified’ descriptions might correspond to default diagnosis descriptions, the information in other free-text fields might state otherwise. Additionally, other free-text fields might include more problems, which therefore implies that diagnoses are missing on the problem list.

#### Error analysis

It is important to note that UnLaTem is meant to identify simple sentences including uncertainty, laterality and temporality using regular expressions, and was never expected to capture all properties. We believe that the simplicity of this rule-based algorithm makes it appealing to apply to modified diagnosis descriptions, especially for developers without training in NLP [21]. Nonetheless, there are more contextual properties to be considered for a more complex version of the algorithm. ContextD, an algorithm adapted from ConText [17] identifies whether a condition is temporal, but also whether terms were negated in clinical Dutch reports and letters [27]. Previous research showed that half of the terms retrieved in clinical reports were negated [61]. Hence, the negation detection module by ConTextD could be considered to add to future editions of UnLaTem. Furthermore, the “missing terms” class of errors showed that there are more variations to describe temporality [17, 62]. For example, by adding variations to describe temporality by including a historical function such as *hypertension in the past* (Dutch: *hypertensie in het verleden*). It is important to note that some terms were purposely not added, such as ‘request’. Terms like ‘request’ are disputable because it has several definitions. For instance, ‘request’ could suggest requests for lab tests to confirm a diagnosis, which implies it is a kind of uncertainty. However, ‘request’ could also mean that healthcare providers request treatments for that specific diagnosis, which suggests it is not an uncertainty.

We also discovered that abbreviations were sometimes misclassified. For example, *AD* means *right ear* but was also used for the pregnancy duration (Dutch: *Amenorroe duur*). Misusing abbreviations could lead to medical errors [63, 64]. The algorithm also misclassified diagnosis descriptions containing values such as *mmol/l*. That is because *l* was one of the trigger items for laterality. Furthermore, the algorithm showed lower performance scores for removal of uncertainty, because some term removals were not discovered in the training set. Additionally, although *possible* (Dutch: *waarschijnlijk* and *mogelijk*) was included in the algorithm, the regular expression only returned True for uncertainty if *possible* occurred at the beginning of the sentence. In the error analysis, we found that *possible* also appeared in the middle of sentences. Extending the rules for existing trigger items for laterality and (removal of) uncertainty could improve the performance of the algorithm even further.

In the third error class, “outside the framework”, we discovered that the algorithm did not take into account the term order of the modified descriptions, though this is needed to extract meaningful information [27]. For instance, if the diagnosis code is *stomach ache* and the modified description is *stomach ache, suspected flu,* the algorithm will flag this as a case of uncertainty, although it was not the *stomach ache* that was uncertain, but the flu. Finally, the annotators sometimes misclassified a modified description as an uncertainty, or they overlooked a laterality. Before the algorithm can be implemented to larger free-text documents, more research is needed to determine variations in describing other contextual properties and the term order.

### Relation to other literature

We compared the results of temporality of UnLaTem and the temporality module of ContextD. Laterality was not included in ContextD. UnLaTem performed better on temporality (recall = 0.97, precision = 0.95) compared to averaged performance rates of the temporality function of ContextD (recall = 0.73, precision = 0.38). It is important to note that there were more trigger items (i.e. *since*) for the temporality function of ContextD than for the temporality function of UnLaTem. Another comparable algorithm is PYContextNLP [21]. PyContextNLP reports among others whether uncertainty is present or absent for a diagnosis in English text. The performance scores for uncertainty of PyContextNLP (recall = 0.94, precision = 0.93) are comparable to the performance scores for uncertainty of UnLaTem (recall = 0.98, precision = 0.90). PyContextSwe is the Swedish version of PyContextNLP, and distinguishes four different classes (definite existence, probable existence, probable negated existence and definite negated existence) [65]. Probable existence is comparable to uncertainty. The performance of probable existence was slightly lower (recall = 0.81, precision = 0.82) than UnLaTem’s performance on uncertainty (recall = 0.98, precision = 0.90). Additionally, UnLaTem included laterality triggers. This means that UnLaTem could also be used to detect patterns of laterality for implementation in context-sensitive user interfaces and identify terms that should be further specified by laterality. One solution could be that when the healthcare provider selects the term *eye infection,* a check-box of ‘left’ and ‘right’ is presented in the design of the EHR system, as we found laterality was commonly added in the modified descriptions. However, no check-box should appear when the healthcare provider selects terms for non-lateralizable concepts, for instance *diabetes.* Additionally, based on the presence of (removal of) uncertainty, laterality or temporality in the modified descriptions, the algorithm could trigger alerts to a decision support system or module to no longer rely on the captured underlying codes. We believe that UnLaTem is therefore an useful addition as it can provide meaningful insights how information is recorded on the problem list.

## Conclusions

An algorithm called UnLaTem was developed to identify contextual properties in Dutch modified diagnosis descriptions, including (removal of) uncertainty, laterality and temporality, which is publicly available for other researchers to be used for further improvement or application within their institutions [66]. Our results indicate that the approach used to develop UnLaTem performs well for identifying the four contextual properties in diagnosis descriptions, which could help improve overall precision of information retrieval and extraction. It thereby provides insights in the correctness of diagnosis descriptions and potential discrepancies, that should be identified before reusing these diagnosis data. However, UnLaTem could be improved with more contextual properties including new trigger terms, extension of the rules and the recognition of the term order, before it can be applied to larger free-text documents in EHR systems. UnLaTem could be implemented eventually in Dutch hospital systems, improving quality of diagnosis data for research and clinical decision support. Finally, although the current algorithm focuses on the Dutch language, the methods to develop and evaluate such an algorithm can be generalized to other languages.

### Supplementary Information

**Additional file 1.** Translation of Figure 1.

**Additional file 2.** Code to determine contextual properties and outcomes per specialty.

## Data Availability

The data are not publicly available as they contain information that could harm the privacy regulations in the Netherlands. The complete overview of outcomes of the algorithm and guidelines used for manual annotation can be requested by ESK. The code used to determine the contextual properties and outcomes per specialty can be found in the Additional R-file or can be downloaded via: https://github.com/evaklappe/UnLaTem (Additional file 2).

## Appendix 1. Recategorization of specialties

In the multicenter dataset (n = 175,210), 62 different specialty names were available (shown in the left column of Table 7). The right column shows the renaming of the specialties. For example, the records for Audiological Centres (Dutch: *audiologische centra*) are combined with audiology (Dutch: *audiologie*).

**Table 7:** Original names of the specialties and the new names of the specialties

Original names	New names
Allergology	Allergology
Anesthesiology	Anesthesiology
Pharmacists	Pharmacists
Audiology	Audiology
Audiological centres	Audiology
Cardiology	Cardiology
Cardio pulmonary surgery	Thoracic surgery
Thoracic surgery	Thoracic surgery
Surgery (Dutch: chirurgie)	Surgery
Dermatology	Dermatology
Diabetes Nurses	Specialized nurses (diabetes, emergency care, oncology, ostomy and unspecified)
Dieticians	Dieticians
Occupational therapists	Occupational and physio therapists
Physio therapists	Occupational and physio therapists
Gastroenterology	Gastric, intestinal and liver diseases
Geriatrics	Geriatrics
Specialized nurse, diabetes	Specialized nurses (diabetes, emergency care, oncology, ostomy and unspecified)
Specialized nurse, oncology	Specialized nurses (diabetes, emergency care, oncology, ostomy and unspecified)
Specialized nurse, ostomy	Specialized nurses (diabetes, emergency care, oncology, ostomy and unspecified)
Gynecology / obstetrics	Obstetrics and Gynaecology
Surgery (Dutch: heelkunde)	Surgery
Intensive care medicine	Intensive care medicine
Internal medicine	Internal medicine
Oral surgery	Oral and maxillo-facial surgery
Ear, nose, throat (ENT)	Ear, nose, throat (ENT)
Pediatrics	Pediatrics
Clinical genetics	Clinical genetics
Lung diseases	Lung diseases
Gastric, intestinal and liver diseases	Gastric, intestinal and liver diseases
Medical microbiology	Medical microbiology
Oral and maxillo-facial surgery	Oral and maxillo-facial surgery
Neurosurgery	Neurosurgery
Neurology	Neurology
Obstetrics and gynecology	Obstetrics, gynecology and women's diseases
Oncology nurses	Specialized nurses (diabetes, emergency care, oncology, ostomy and unspecified)
Ophthalmology	Ophthalmology
Optometrist	Ophthalmology
Orthopedics	Orthopedics
Orthoptist	Ophthalmology
Others: general practitioner	Others: general practitioner
Others: emergency care	Emergency care
Others: sports medicine	Sports medicine
Others	Others
Physician Assistant	physician assistant
Plastic surgery	Plastic surgery
Psychiatry	Psychiatry
Psychologists	Psychologists
Psychologists, unspecified	Psychologists
Radiology	Radiology
Radiotherapy	Radiotherapy
Rheumatology	Rheumatology
Rehabilitation	Rehabilitation
Emergency care	Emergency care
Sports medicine	Sports medicine
Dentists: general practitioner	Dentists
Dentist specialists oral diseases and oral surgery	MKA surgery
Urology	Urology
Obstetricians, authorized for ultrasound	Obstetricians (authorized for ultrasound and unspecified)
Obstetricians, unspecified	Obstetricians (authorized for ultrasound and unspecified)
Specialized nurse, emergency care	Specialized nurses (diabetes, emergency care, oncology, ostomy and unspecified)
Nurse, unspecified	Specialized nurses (diabetes, emergency care, oncology, ostomy and unspecified)
Women’s diseases	Obstetrics, gynecology and women's diseases

## Appendix 2. Performance formulas

### 2A: Recall, specificity, prevalence and performance formulas

For the calculation of the recall (or sensitivity), specificity and overall performance, we used confusion matrices similar to Table 8.

**Table 8:** Confusion matrix example

Outcome of the algorithm	Golden standard: the manual annotated datasets	
	Yes	No
Yes	True Positive (TP)	False Positive (FP)
No	False Negative (FN)	True Negative (TN)

We categorized four possible outcomes; True Positives (TP), False Positives (FP), False Negatives (FN) and True Negatives (TN). TP is a correctly identified description, FP is an incorrectly identified description, FN is an incorrectly denied description and TN is a correctly denied description.

With these numbers, the performance of the algorithm UnLaTem can be assessed by:

- Actual prevalence, or the proportion of records with a definitive modification type: TP + FN / (TP + FN + FP + TN)
- Precision: TP / (TP + FP)
- Recall: TP / (TP + FN)
- Specificity: TN / (FP + TN)
- Standard error (SE), where n = sample size, such that:
  - SE recall: √ TP*FN / (TP + FN)^3
  - SE specificity: √ FP*TN / (FP + TN)^3
  - SE proportion of patients with a definitive property (prevalence): √ (TP + FP)(FN + TN)/ (TP + FN + FP + TN)^3
  - SE precision: √ p * (1-p)/n, where p = precision of the internal or multicenter validation set
- Weighted means, where p = recall, specificity, prevalence or precision of the internal validation set (1) or the multicenter validation set (2):
  $$\left( {{\text{p}}/{\text{SE}}_{{{\text{P1}}}}^{2} + {\text{ p}}/{\text{SE}}_{{{\text{P2}}}}^{2} } \right) \, / \, \left( { \, 1/{\text{ SE}}_{{{\text{P1}}}}^{2} + \, 1/{\text{ SE}}_{{{\text{P2}}}}^{2} } \right)$$
  - Note that for the weighted means of the recall, specificity and prevalence we added 0.5 to all counts

### 2B: Prediction formulas

Here, “the property type” refers to uncertainty, laterality, temporality or removal of uncertainty.

- The estimated number of descriptions that will truly have the property type (D_true_):
  - Weighted mean of the prevalence * number of modified descriptions (175,210)
    - Uncertainty: 19,240
    - Laterality: 47,932
    - Temporality: 21,663
    - Removal of uncertainty: 2,140
- The number of descriptions that will be recognized by the algorithm to have the property type (A_true_):
  - D_true_ * weighted mean of the recall
    - Uncertainty: 18,869
    - Laterality: 47,880
    - Temporality: 21,091
    - Removal of uncertainty: 1,933
- The number of descriptions that will truly NOT have the property type (D_false_):
  - Number of modified descriptions (175,210) − D_true_
    - Uncertainty: 155,970
    - Laterality: 127,278
    - Temporality: 153,547
    - Removal of uncertainty: 173,070
- Wrongly categorized descriptions (A_false_)
  - (1-Specificity) * D_false_
    - Uncertainty: 2,110
    - Laterality: 1,832
    - Temporality: 1,083
    - Removal of uncertainty: 341
- Total descriptions determined by the algorithm to have the property type (A_total_):
  - A_false_ + A_true_
    - Uncertainty: 20,979
    - Laterality: 49,713
    - Temporality: 22,174
    - Removal of uncertainty: 2,275
- Estimated percentage of descriptions to have the property type (%):
  - 100 * (1 − A_false_/A_total_)
    - Uncertainty: 89.9
    - Laterality: 96.3
    - Temporality: 95.1
    - Removal of uncertainty: 85.0

### 2C: Actual prevalence, using the Rogan–Gladen estimator [54]

- Actual prevalence = Apparent prevalence + (weighted mean of the specificity − 1) / weighted mean of the specificity + (weighted mean of the recall − 1)
  - Uncertainty (apparent prevalence: 16,779): 17,347
  - Laterality (apparent prevalence: 54,081):54,931
  - Temporality (apparent prevalence: 16,582): 17,156
  - Removal of uncertainty (apparent prevalence: 1,991):2,208

## *Confusion matrices per modification type for the internal (n* = *980) and multicenter (n* = *996) validation sets.*

### Internal validation

See Tables 9, 10, 11 and 12.

**Table 9:** The uncertainty confusion matrix of the internal validation set

Uncertainty n = 980	Golden standard	
	Annotators predicted: Yes	Annotators predicted: No
Algorithm predicted: Yes	TP = 116	FP = 13
Algorithm predicted: No	FN = 1	TN = 850

**Table 10:** The laterality confusion matrix of the internal validation set

Laterality n = 980	Golden standard	
	Annotators predicted: Yes	Annotators predicted: No
Algorithm predicted: Yes	TP = 248	FP = 7
Algorithm predicted: No	FN = 0	TN = 725

**Table 11:** The temporality confusion matrix of the internal validation set

Temporality n = 980	Golden standard	
	Annotators predicted: Yes	Annotators predicted: No
Algorithm predicted: Yes	TP = 162	FP = 4
Algorithm predicted: No	FN = 4	TN = 810

**Table 12:** The removal of uncertainty confusion matrix of the internal validation set

Removal of uncertainty n = 980	Golden standard	
	Annotators predicted: Yes	Annotators predicted: No
Algorithm predicted: Yes	TP = 9	FP = 6
Algorithm predicted: No	FN = 0	TN = 965

### Multicenter validation

See Tables 13, 14, 15 and 16.

**Table 13:** The uncertainty confusion matrix of the multicenter validation set

Uncertainty n = 996	Golden standard	
	Annotators predicted: Yes	Annotators predicted: No
Algorithm predicted: Yes	TP = 88	FP = 11
Algorithm predicted: No	FN = 14	TN = 883

**Table 14:** The laterality confusion matrix of the multicenter validation set

Laterality n = 996	Golden standard	
	Annotators predicted: Yes	Annotators predicted: No
Algorithm predicted: Yes	TP = 290	FP = 20
Algorithm predicted: No	FN = 1	TN = 685

**Table 15:** The temporality confusion matrix of the multicenter validation set

Temporality n = 996	Golden standard	
	Annotators predicted: Yes	Annotators predicted: No
Algorithm predicted: Yes	TP = 91	FP = 10
Algorithm predicted: No	FN = 3	TN = 892

**Table 16:** The removal of uncertainty confusion matrix of the multicenter validation set

Removal of uncertainty n = 996	Golden standard	
	Annotators predicted: Yes	Annotators predicted: No
Algorithm predicted: Yes	TP = 8	FP = 1
Algorithm predicted: No	FN = 6	TN = 981

## Appendix 4. Specialties and contextual properties

See Table 17.

**Table 17:** Specialties and properties in n and % in the multicenter dataset (n = 175,210). Ordered by descending number of modified descriptions. Shown is the actual prevalence, which was determined using the Rogan–Gladen estimator

Specialties	Modified descriptions, n	Uncertainty, n (%)	Laterality, n (%)	Temporality, n (%)	Removal of uncertainty, n (%)
Total	175,210	17,347 (9.9)	54,931 (31.4)	17,156 (9.8)	2208 (1.3)
Internal medicine	30,457	2326 (7.6)	4254 (14.0)	4822 (15.8)	562 (1.8)
Pediatrics	18,776	1818 (9.7)	2018 (10.7)	1475 (7.9)	238 (1.3)
Ophthalmology	16,901	332 (2.0)	13,471 (79.7)	605 (3.6)	253 (1.5)
Neurology	12,375	1990 (16.1)	4451 (36.0)	798 (6.4)	145 (1.2)
Cardiology	10,766	460 (4.3)	983 (9.1)	351 (3.3)	232 (2.2)
Surgery	9418	298 (3.2)	4626 (49.1)	1255 (13.3)	25 (0.3)
Orthopedics	8279	192 (2.3)	7355 (88.8)	1005 (12.1)	1 (0.01)
Emergency care	7796	235 (3.0)	3304 (42.4)	2357 (30.2)	21 (0.3)
Ear, nose, throat (ENT)	7349	652 (8.9)	2908 (39.6)	366 (5.0)	8 (0.1)
Lung diseases	6196	611 (9.9)	676 (10.9)	298 (4.8)	80 (1.3)
Other	6019	288 (4.8)	1591 (26.4)	725 (12.0)	36 (0.6)
Clinical genetics	5655	5287 (93.5)	238 (4.2)	2 (0.04)	27 (0.5)
Obstetrics, gynecology and women's diseases	4914	226 (4.6)	315 (6.4)	185 (3.8)	114 (2.3)
Anesthesiology	3767	192 (5.1)	640 (17.0)	456 (12.1)	8 (0.2)
Rheumatology	3158	243 (7.7)	435 (13.8)	353 (11.2)	35 (1.1)
Gastric, intestinal and liver diseases	3043	197 (6.5)	174 (5.7)	529 (17.4)	33 (1.1)
Plastic surgery	2918	64 (2.2)	1636 (56.1)	44 (1.5)	1 (0.03)
Geriatrics	2889	359 (12.4)	650 (22.5)	118 (4.1)	25 (0.9)
Neurosurgery	2135	256 (12.0)	879 (41.2)	270 (12.6)	19 (0.9)
Dermatology	2047	397 (19.4)	427 (20.9)	48 (2.3)	10 (0.5)
Radiotherapy	1856	83 (4.5)	909 (49.0)	53 (2.9)	6 (0.3)
Urology	1306	35 (2.7)	486 (37.2)	58 (4.4)	49 (3.8)
Others: general practitioner	1223	42 (3.4)	222 (18.2)	19 (1.6)	4 (0.3)
Audiology	1127	4 (0.4)	133 (11.8)	0 (0.0)	0 (0.0)
Thoracic surgery	1065	68 (6.4)	141 (13.2)	87 (8.2)	3 (0.3)
Specialized nurses (diabetes, emergency care, oncology, ostomy and unspecified)	749	12 (1.6)	299 (39.9)	146 (19.5)	5 (0.7)
Rehabilitation	724	23 (3.2)	453 (62.6)	102 (14.1)	0 (0.0)
Sports medicine	638	0 (0.0)	31 (4.9)	0 (0.0)	0 (0.0)
Psychiatry	486	23 (4.7)	27 (5.6)	36 (7.4)	0 (0.0)
Obstetricians (authorized for ultrasound and unspecified)	354	21 (5.9)	9 (2.5)	9 (2.5)	1 (0.3)
Physician assistant	225	10 (4.4)	160 (71.1)	0 (0.0)	0 (0.0)
Oral and maxillo-facial surgery	207	1 (0.5)	101 (48.8)	4 (3.4)	1 (0.9)
Intensive care	138	9 (6.5)	24 17.4)	3 (2.2)	0 (0.0)
Radiology	69	4 (5.8)	35 (50.7)	2 (2.9)	0 (0.0)
Medical microbiology	59	6 (10.2)	1 (1.7)	0 (0.0)	49 (83.1)
Allergology	55	14 (25.5)	5 (9.1)	0 (0.0)	0 (0.0)
Dentists	53	1 (1.9)	3 (5.7)	1 (1.9)	0 (0.0)
Occupational and physio therapists	11	0 (0.0)	7 (63.6)	0 (0.0)	0 (0.0)
Psychologists	5	0 (0.0)	1 (20.0)	0 (0.0)	0 (0.0)
Pharmacists	1	0 (0.0)	0 (0.0)	0 (0.0)	0 (0.0)
Dieticians	1	0 (0.0)	0 (0.0)	0 (0.0)	0 (0.0)

## Abbreviations

UMC: Amsterdam University Medical Center
DT: Diagnosis thesaurus
EHR: Electronic health record
UnLaTem: Uncertainty, laterality, temporality
NLP: Natural language processing
