# Supplementary material for: Contextual property detection in Dutch diagnosis descriptions for uncertainty, laterality and temporality
Source: BMC Med Inform Decis Mak. 2021 Apr 7;21:120. doi: 10.1186/s12911-021-01477-y (PMC8028823; doi:10.1186/s12911-021-01477-y)
Supplement: Supplementary file 1 — Additional file 1. Translation of Figure 1. [file 12911_2021_1477_MOESM1_ESM.docx]

**Supplemental file – translation of Figure 1.**

**Uncertainty**

- Verdenk = suspect
- Beoordeel = judge
- Onderzoe = research
- Erfelijk = heritable
- Screen = screening
- Potentie = potential
- Niet bevestigd = not confirmed
- Niet zeker = not certain
- Waarschijnlijk = probably
- Mogeiljk = possible
- Controle = check
- Vermoed = presumably
- Analyse = analysis
- Vraag = ask
- Advies = advice
- Wrs = abbrevation for ‘waarschijnlijk’, similar to ‘probably’

**Temporality**

- Januari = January
- Februari = February
- Maart = March
- April = April
- Mei = May
- Juni = June
- Juli = July
- Augustus = August
- September = September
- Oktober = October
- November = November
- December = December

**Laterality**

- Rechts / rechter / re = right / ri
- Links / linker / le = left / le
- Sinister / sinistra = Latin names for left-hand side
- Dexter / dextra = Latin names for right-hand side
- Beide / beiderzijds = both / both sides
- Unilatera = unilateral or one-sided
- Bilatera / bifrontaal = bilateral or two-sided
